# Antimicrobial Activity of Probiotic Bacteria Isolated from Plants: A Review

**DOI:** 10.3390/foods14030495

**Published:** 2025-02-04

**Authors:** Anshul Sharma, Hae-Jeung Lee

**Affiliations:** 1 Department of Food and Nutrition, College of BioNano Technology, Gachon University, Seongnam-si 13120, Republic of Korea; anshul.silb18@gmail.com; 2 Institute for Ageing and Clinical Nutrition Research, Gachon University, Seongnam-si 13120, Republic of Korea; 3 Department of Health Sciences and Technology, Gachon Advanced Institute for Health Sciences and Technology, Gachon University, Incheon 21999, Republic of Korea

**Keywords:** antibacterial, antifungal, bacteriocin, lactic acid bacteria, organic acid, pathogens

## Abstract

Lactic acid bacteria (LAB) constitute a heterogeneous group of bacteria isolated from fermented foods, animals, plants, and mammalian guts, with many health-promoting properties. Probiotics with antagonistic properties against human pathogens and foodborne bacteria have garnered significant attention from the scientific fraternity. A dedicated review focusing on plant-derived probiotic bacteria and their antagonistic properties has not been comprehensively reviewed. Thus, this review aimed at providing an overview of LAB isolates derived from several unconventional sources such as fruits, seeds, fruit pulp, leaves, roots, vegetables, grasses, and flowers and with their antibacterial, antifungal, and antiviral properties. This paper reviewed the antimicrobial properties of different genera, *Lactobacillus*, *Leuconostoc*, *Weissella*, *Enterococcus*, *Pediococcus*, *Bacillus*, and *Fructobacillus*, their postbiotics, and paraprobiotics. Several important mechanisms, including the secretion of bacteriocins, bacteriocin-like substances, reuterin, organic acids (lactic and acetic), peptides, exopolysaccharides, and hydrogen peroxide, have been attributed to their antimicrobial actions against pathogens. However, their precise mode of action is poorly understood; hence, further research should be conducted to reveal detailed mechanisms. Finally, the review discusses the summary and future implications. Given the significance, LAB and derived antimicrobial compounds can potentially be exploited in food preservation and safety or for medicinal applications after evaluating their safety.

## 1. Introduction

Lactic acid bacteria (LAB) are Gram-positive, non-motile bacteria with rod and coccus shapes, naturally present in several food and food products and also in non-food-related sources such as soil, plants, and the guts of mammals [1,2]. These microorganisms grow in microaerophilic or anaerobic environments and are acid-tolerant in nature [3]. A common characteristic of them is to produce lactic acid as their sole product during the fermentation of carbohydrates. Probiotic LAB are generally regarded as safe (GRAS), and according to the experts from the International Scientific Association for Probiotics and Prebiotics (ISAPP), they are redefined as “live microorganisms that, when administered in adequate amounts, confer a health benefit on the host” [4]. Examples of the best-known probiotic strains belong to the genera *Bifidobacterium*, *Lactobacillus*, *Lactococcus*, *Streptococcus*, *Weissella*, *Enterococcus* the fungus genus *Saccharomyces*, and the spore-forming genera *Bacillus*, *Sporolactobacillus*, and *Brevibacillus* [5,6,7]. These probiotic isolates from diverse sources can be classified as either conventional or next-generation, depending on their origin, development, safety, strain specificity, application, and regulation [8,9]. Examples of next-generation probiotics include *Akkermansia*, *Bacteroides*, *Christensenella*, *Faecalibacterium*, *Prevotella*, *Roseburia*, and others.

As per the 2023 statistics from Grand View Research, the probiotic market is expected to witness a rise in annual growth by 14.1% from 2024 to 2030, with a USD 220.2 billion revenue forecasted in 2030. In 2024, the market size value for probiotics was estimated to be USD 99.97 billion [10]. Probiotics offer an attractive option for the development of functional foods, as they are documented to have many health-promoting properties, including improving gastrointestinal health, enhancing host immunity, improving obesity and cardiometabolic health, gut microbiota regulation, the prevention and treatment of diabetes and cancer, and improving neurodegenerative disease [6,11,12,13]. Besides probiotics, other functional attributes represent the use of non-viable organisms known as paraprobiotics, plant secondary metabolites as prebiotics, and extracellular metabolites known as postbiotics [14,15,16]. In general, marketed oral probiotics originate from human sources; nevertheless, foods, especially naturally fermented ones, also include native probiotic microbes [17].

**Figure 1:**
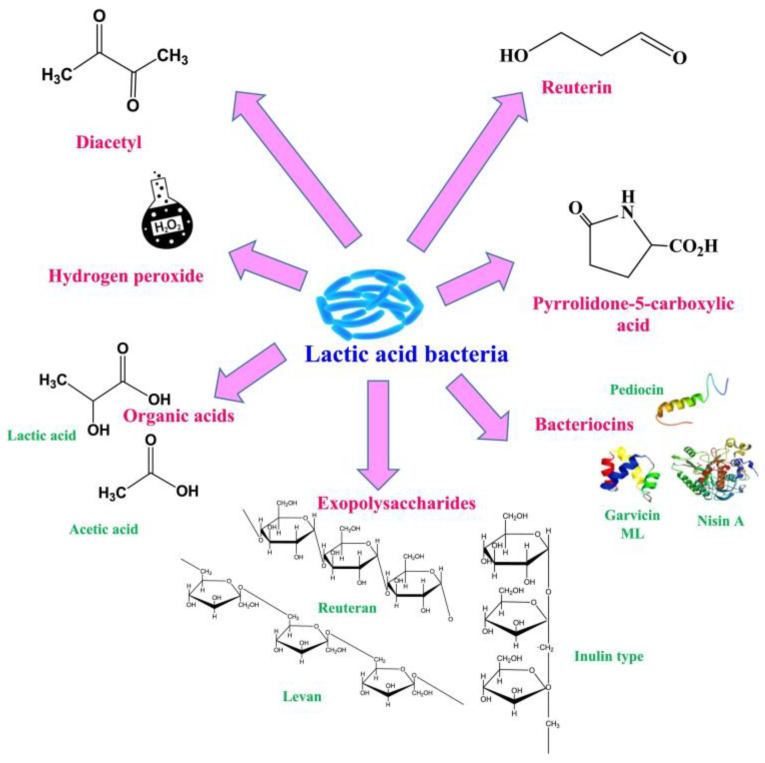
The illustration depicts the secretion of some bioactive metabolites by lactic acid bacteria. Reproduced with kind permission from [21] copyright Elsevier 2024.

These beneficial microbes predominantly play an important role in the safety, shelf-life extension, flavor, and aroma of several products, proteolytic activity, and the production of vitamins, polysaccharides, and antimicrobial compounds [18,19,20]. The assessment of antimicrobial activity against pathogens is a crucial factor in the selection of potential probiotic strains. This functionality of LAB acts as a checkpoint for the food product’s integrity by preventing food spoilage and ensuring the consumers’ safety. Probiotics LAB are known to secrete a variety of bioactive compounds (Figure 1); most of them have been reported to act as antimicrobials [21].

It is widely acknowledged that the antibacterial activity of probiotic bacteria is a significant probiotic characteristic. However, the antimicrobial property of probiotic LAB is derived from strains that are isolated and purified from fermented foods and products. Plants provide a natural home for numerous microbes, including beneficial probiotics. The information on plant-derived probiotic LAB and their antimicrobial properties is scarce and not compiled systematically. Previous lustrum works focused on the antimicrobial activity of probiotic bacteria are those of Silva et al. [20], Agriopoulou et al. [22], Fijan [23], and Ibrahim [24]. In this review, we compiled data related to probiotic bacteria isolated from plants and their different parts and assessed their antimicrobial activity. As per true to our knowledge, no previous review has been published on this subject.

## 2. Probiotic Bacteria and Antimicrobial Activity

Antimicrobial activity against pathogens is an important feature. Probiotics modulate gastrointestinal disorders by demonstrating their antimicrobial and anti-adhesion effects against different pathogens [25]. Secretion of antimicrobial substances depends upon the composition of the culture medium and the genetic makeup and physiology of the strain [26]. The antimicrobial activity of LAB can be classified as direct and indirect [27]. Direct action is mostly initiated by bacterial metabolites that eliminate or suppress the growth of pathogens or obstruct their adhesion, colonization, and biofilm formation, whereas indirect action depends on the interaction between the host and probiotic bacteria. Different strains of probiotic bacteria release active metabolites (primary and secondary) that result in broad-spectrum activity against pathogenic microorganisms [27].

### 2.1. Bacteriocin and Bacteriocin-like Inhibitory Substances

These active compounds are further categorized into two types: bacteriocins and non-bacteriocin compounds [2]. Originally discovered by Gratia in 1925 [28], while the word bacteriocin was coined by Jacob et al. [29], defined as small proteins or peptides synthesized using ribosomal machinery released outside the cell that are adept at killing bacteria, and sometimes they display antifungal activity, described in detail elsewhere [21]. Moreover, Gram-positive LAB, endospore-forming *Bacillus* spp., are also documented to be major producers of bacteriocins. Bacteriocins could work against multi-drug-resistant bacteria and improve the action of antibiotics [30]. Furthermore, bacteriocins have been documented to display antiviral [31] and antileishmanial activity [32]. Bacteriocins are usually active against strains closely related to the producing strain, though there are cases of broader spectrum action of bacteriocins. In general, the application of bacteriocins has been documented in the food industry (food preservation, food packaging, aquaculture, and seafood), the pharmaceutical industry (oral care, skin care, cancer therapy, and antiviral), the agricultural industry, and in commercial products [33]. Many studies have reported that the release of bacteriocin by probiotic LAB could protect the host from pathogen invasion. For example, the viability of *Helicobacter pylori* could be reduced by bacteriocin production by *Lactobacillus acidophilus* [34]. Oh et al. [35] elucidated the antimicrobial activity of a bacteriocin produced by *Lactococcus* sp. HY 499 against *Streptococcus pyogenes*, *Staphylococcus epidermidis*, *Staphylococcus aureus*, and *Propionibacterium acnes*. Among all, *Staphylococcus aureus* exhibited double the sensitivity to the bacteriocin compared to others. The authors proposed utilizing the bacteriocin produced by HY 449 as an antimicrobial agent in cosmetic formulations [35]. Similarly, *Bifidobacterium animalis* subsp. *lactis* was shown to secrete a bacteriocin-like inhibitory substance that could inhibit the growth of *Listeria monocytogenes* [36].

### 2.2. Non-Bacteriocin Types

The second category includes various non-bacteriocin compounds, such as organic acids (e.g., lactic, citric, acetic, fumaric, and malic acid), hydrogen peroxide (H_2_O_2_), diacetyl, acetaldehyde, acetoin, exopolysaccharides (EPS), reuterin, carbon dioxide (CO_2_), reutericyclin, ethanol, ammonia, and antifungal compounds (cyclic dipeptides, phenyl-lactate, 3-hydroxy fatty acids, propionate, and hydroxyphenyl-lactate) [22,37] (Figure 1). Lactic and acetic acids are the most important and thoroughly researched organic acids. Lactic acid is a primary metabolite generated by all LAB. Previously, the inhibitory effects of lactic acid and acetic acid have been demonstrated against the growth of *Aspergillus flavus*. Minimum inhibitory concentration (MIC) value is defined as the lowest concentration of an antimicrobial agent at which visible growth of a microorganism is completely inhibited. As per this study, the MIC value for lactic acid was observed to be tenfold higher than the MIC values of acetic acid. In the synergistic way, the acid mixtures reduced the concentration of every acid necessary for fungal inhibition compared to MIC values [38].

Acetic acid is exclusively generated by heterofermentative LAB [39]. The accumulation of acids results in a decrease in pH, which not only demonstrates broad-spectrum antagonistic activity but also promotes the growth of beneficial microbes. Acetic acid reported to have stronger antagonistic activity than lactic acid [39]. It has been reported that the acetate produced by *Bifidobacterium* increases intestinal protection mediated by epithelial cells and thereby protects the host against infection by enterohemorrhagic *Escherichia coli* O157:H7 [40]. Another in vitro study indicated that lactic acid production by *Lactobacillus crispatus* JCM 5810 was identified as a contributing factor responsible for the decrease in the total number of *Campylobacter jejuni* colonized chickens and lowered microbial load [41]. These studies suggest the potential antagonistic role of organic acids produced by probiotic lactobacilli.

Heterofermentative LAB species comprise flavoprotein oxidase enzymes that facilitate the reduction of oxygen, resulting in the production of H_2_O_2_ in the absence of intracellular catalase, pseudocatalase, or peroxidase [24]. The antimicrobial activity of H_2_O_2_ is attributed to the production of oxidative species such as superoxide ions (O_2_^−^) and hydroxyl radicals (OH) via the Fenton reaction, which disrupts vital biological components, including DNA and proteins, culminating in microbial cell death [42]. A previous study has reported the selection of H_2_O_2_-producing lactobacilli from a group of 72 strains isolated from the vaginal microbiota of cattle. Among all, two strains of *Lactobacillus gasseri* (CRL1421 and CRL1412), with probiotic properties, demonstrated the ability to produce H_2_O_2_, detected by the plate colorimetric method. The strain CRL1421, the superior H_2_O_2_ producer, showed inhibition of *Staphylococcus aureus* due to the release of H_2_O_2_ and lactic acid [43].

Similarly, heterofermentative LAB release CO_2_ that is reported to exhibit the antimicrobial activity [39]. Diacetyl (2,3-butanodione) is a volatile compound that operates by prohibiting vital enzymes in target microorganisms, which alter their catalytic functions [24,44,45]. Its production was reported at yogurt formation or ripening of cheese by citrate utilizing LAB [46]. Diacetyl, acetoin, and acetaldehyde generated by heterofermentative LAB contribute to the extension of shelf life in certain foods by inhibiting the proliferation of spoilage Gram-positive, Gram-negative bacteria, and yeasts [47]. Diacetyl is reported to be more effective against Gram-negative bacteria, molds, and yeasts than Gram-positive bacteria [48].

*Limosilactobacillus reuteri* produces reuterin (3-hydroxypropionaldehyde), a non-protein, broad-spectrum antimicrobial substance that inhibits numerous Gram-negative and Gram-positive bacteria by deactivating the important enzyme ribonucleotide reductase [24,49,50]. Reuterin also acts as a potent antimicrobial agent against fungi, yeast, and protozoa [51,52]. Reports showed that treating acidified dairy products with reuterin and diacetyl had promising antimicrobial effects on controlling food pathogens [45].

Phenyl lactic acid, or 2-hydroxy-3-phenyl propionic acid, is a metabolic substance secreted by LAB and is a major keystone of the antifungal activity [53,54]. Likewise, 3-hydroxy fatty acids display antifungal activity and are documented to be produced by *Lactiplantibacillus plantarum* [55]. Polysaccharides, or glycans, are universally found in the cell walls of Lactobacilli. These cell surface structures differ in terms of sugar compositions, types of linkages, polysaccharide branching, configuration, molecular weight, and specific modifications like acetylations, phosphorylations, etc. [56]. LAB EPS are documented to possess antimicrobial activity. The comprehensive explanations pertaining to the antibacterial, antiviral, antifungal, and anti-biofilming actions of EPS derived from LAB have been described elsewhere [57].

### 2.3. Indirect Antimicrobial Activity

**Figure 2:**
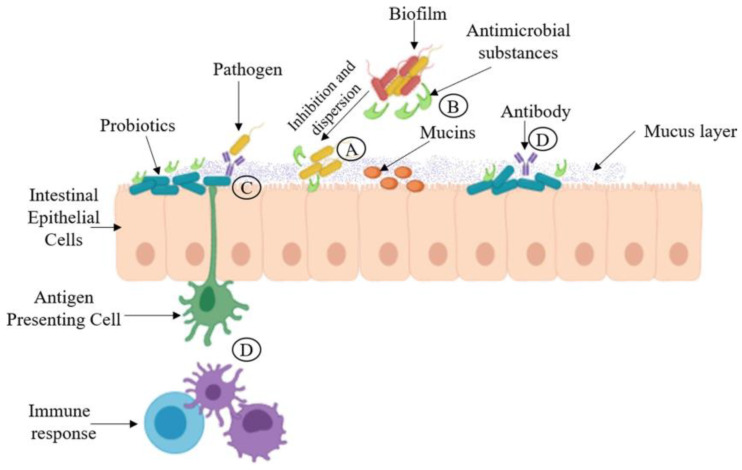
The illustration shows the mechanism of action of probiotics. (A) Competitive exclusion of pathogenic microorganisms. (B) Production of antimicrobial substances. (C) Increased adhesion to the intestinal mucosa and improvement of the epithelial barrier. (D) Stimulation of the immune system. Reproduced with kind permission from [20] copyright Elsevier 2024.

Concerning indirect antimicrobial activity, probiotics include different strategies that aid in their inhibitory activity against pathogens. These include (1) increased epithelial barrier, (2) increased adhesion to the intestinal mucosa and inhibition of pathogen microbial adhesion, (3) competitive exclusion of pathogenic microorganisms, (4) production of antimicrobial substances, and (5) modulation of the immune system [58]. Figure 2 shows a schematic representation of how these mechanisms occur in the intestinal mucosa.

Probiotic bacteria can reduce pathogenic growth and colonization via competition with pathogens for nutrients and attachment sites, thus diminishing the risk of infections. Probiotics impede the adhesion of enteropathogens to the intestinal surface and subsequent infection by competing for binding sites [59]. For example, different strains of *Lactobacillus* have been established to follow a competitive exclusion strategy by displacing attachments of enteropathogens as assessed through competitive adhesion assays. Precisely, compared to the control (adhesion of pathogens in the absence of lactobacilli), *Lacticaseibacillus rhamnosus* GG, *Lactiplantibacillus plantarum* strains (CS23 and CS24.2), and *Lactobacillus delbrueckii* M. were able to effectively antagonize the adhesion of *Salmonella enterica* serovar Typhi and *Escherichia coli* O26: H11 (EPEC) to mucin. This study highlights the remarkable role of the elongation factor Tu in the adhesion of lactobacilli and inhibition of pathogens [60]. Another interesting in vivo study, firstly, investigated the role of mucus adhesion protein from *Lactiplantibacillus plantarum* 423 in competitive exclusion of *Listeria monocytogenes* EGDe in the gastrointestinal tract of mice [61]. Collagen-binding protein can also work as an important factor in the competitive exclusions of pathogens. For example, collagen-binding protein from *Lactobacillus fermentum* RC-14 demonstrated high anti-adhesive properties against *Enterococcus faecalis* 1131 (an uropathogen) [62].

More recently, biofilm formation by LAB as an effective antimicrobial strategy against pathogens has garnered the significant attention of the scientific community [63]. The process by which LAB biofilms fight against harmful bacteria is based on the competitive exclusion principle, which has been documented in two separate ways: the Jameson effect and spatial competition. In the former, both LAB and pathogenic bacteria compete for resources, and LAB win by developing bio-films that block nutritional intakes, resulting in cell death of pathogenic bacteria. In nutrient spatial competition, metabolically active LAB outcompetes pathogenic bacteria for nutrients by forming biofilm. Formation of biofilm by LAB restricts pathogens at its bottom layer of biofilm; due to a shortage of nutrients, pathogen growth is restricted [63].

Apart from that, it is well understood that the gut barrier is an important defense system that maintains the critical balance of the intestine [64]. This barrier’s compromised integrity causes dysfunction in absorptive capacity, chemical and microbial hazards, as well as a variety of immunological and inflammatory illnesses [65]. Evidence suggests that certain probiotic *Lactobacillus* strains may impede pathogen adherence and safeguard the integrity of cell junctions and lead to the enforcement of the barrier function [66]. Moreover, it has been reported that the protective effects of probiotics against gut barrier dysfunction are due to the modulating effects of Toll-like receptor 2 [67]. Another indirect mechanism involves host immunomodulation whereby probiotic LAB help to synergistically activate both innate and adaptive immune responses and exert their effects by activating natural killer cells and macrophages, secreting cytokines, and enhancing the production of bioactive components [68,69]. Evidence suggests that the pretreatment with probiotic strains, *Lacticaseibacillus rhamnosus* GG, *Lacticaseibacillus* rhamnosus KLDS, *Lacticaseibacillus* casei IMAU60214, and *Lactobacillus helveticus* IMAU70129, augmented phagocytosis and the antibacterial action of macrophages against *Staphylococcus aureus*, *Salmonella* Typhimurium, and *E. coli* [70].

## 3. Literature Search Strategy

Comprehensive search was conducted using online search tools such as Google Scholar, Embase, PubMed, Scopus, and Science Direct and articles published between 2005 and 2024 for selected further for completing this review paper. Related keywords such as ‘lactic acid bacteria’, ‘probiotics’, ‘LAB-derived from plants’, ‘plant parts’ ‘antimicrobial activity’, ‘antagonistic activity’, ‘antibacterial’, ‘antifungal’, ‘antiviral’, ‘bacteriocin’, ‘exopolysaccharides’, ‘peptides’, ‘bacteriocin-like substances’, ‘*Lactobacillus*’, ‘*Leuconostoc*’, *Weissella*’, ‘*Pediococcus*’, ‘*Bacillus*’, ‘*Enterococcus*’, ‘*Fructobacillus*’, and their synonyms were used with ‘OR’ and ‘AND’ in the literature search. Unpublished papers and articles published in languages other than English were excluded. Furthermore, research papers on the antimicrobial property of LAB derived from fermented foods and papers pertaining to plant-derived LAB against plant pathogens were excluded. This review did not follow a systematic review process; therefore, the exact number of publications identified in each literature search was not recorded. This constitutes a limitation in this work. The amended LAB nomenclature has been followed.

## 4. Antimicrobial Activity of Probiotic Bacteria

The antagonistic activity of LAB and probiotic bacterial strains against food spoilage microbes and human pathogens is playing an important role in food preservation and also in inhibiting the growth of undesirable pathogens in the gut microbiota by interfering with them. The antimicrobial activity of different probiotic strains has been described in the following section.

### 4.1. Lactobacillus

Lactobacilli are non-spore-forming, Gram-positive rods that share a large proportion of the normal human bacterial flora and are GRAS. Lactobacilli are reported to have many health-promoting activities, including anti-obesity, immunomodulation, anti-inflammatory, anti-allergic, regulating carbohydrate metabolism, and insulin resistance [71]. Previous review papers have meticulously detailed the antimicrobial, antiviral, antifungal, and anticandidal properties of Lactobacilli [27,72,73]. Considering the importance, a large number of studies are exploring the potential antimicrobial properties of Lactobacilli with implications for the biotechnology sector aimed at enhancing animal and human health [74]. The following section narrates the antimicrobial activities of LAB strains isolated from unconventional plant sources along with their mechanisms of action.

Garcia et al. [75] screened and isolated fifty LAB from fruit pulp processing of *Annona muricata* L. (soursop), *Malpighia glabra* L. (Barbados cherry), *Ananas comosus* L. (pineapple), *Mangifera indica* L. (mango), and *Fragaria vesca* L. (wild strawberry) identified based on 16S rRNA gene sequencing and matrix-assisted laser desorption/ionization–time of flight mass spectrometry. Among all, five LAB strains (Table 1), *Lactiplantibacillus plantarum* 49, *Levilactobacillus brevis* 59, *Lacticaseibacillus paracasei* 108, *Lactiplantibacillus pentosus* 129, and *Limosilactobacillus fermentum* 111, were evaluated for their probiotic and antagonistic activity. Using spot agar and well-diffusion assays, the zone of inhibition (ZOI) of the antagonistic activity of selected five LAB strains ranged from 5 to 10.8 mm and from 1.5 to 3.5 mm for *Salmonella* Enteritidis; from 6 to 8.5 mm and from 2.0 to 3.0 mm for *Salmonella* Typhimurium; from 4 to 8.2 mm and from 2.0 to 4.0 mm for *Listeria monocytogenes*; from 4.5 to 8.5 mm and from 1.5 to 4.5 mm for *E. coli*; and from 4.0 to 9.0 mm and from 2.8 to 4.0 mm for *S. aureus,* respectively. Among all, strains 59 and 111 did not exhibit an inhibitory effect on *S. aureus* [75]. This study suggests that antagonistic activity can be affected by the LAB strain, incubation time, composition of the medium, and evaluation method.

In continued research, seven strains of *Limosilactobacillus fermentum* (56, 60, 139, 141, 250, 263, and 296), one strain each of *Lactiplantibacillus plantarum* 53, and *Lacticaseibacillus paracasei* 106, were further selected and evaluated for antagonistic activity. Using spot agar and well-diffusion assays, the ZOI of all selected nine LAB strains ranged from 9 to 14.0 mm and from 2.0 to 3.5 mm for *Salmonella* Enteritidis; from 6 to 11 mm and from 2.0 to 4.0 mm for *Salmonella* Typhimurium; from 4 to 10 mm and from 2.0 to 4.0 mm for *L. monocytogenes*; and from 4.0 to 12.0 mm and from 2.0 to 3.0 mm for *E. coli*, respectively. Except for 106, 141, 250, and 296 strains, the ZOI for spot agar and well-diffusion assays ranged from 4.0 to 11 mm and from 2.0 to 5.0 mm for *S. aureus*. Stronger antagonistic activity was observed for *L. plantarum* 53 and *L. fermentum* 60. In both studies, spot assay showed a higher ZOI against all indicator strains than well diffusion assay. Despite good antagonistic activity against selected pathogens, both studies suffer from a limitation that the nature of the antimicrobial compound(s) was not evaluated [76].

Exopolysaccharides are essential postbiotic components with important applications in the food and health industries, including antioxidant, antibacterial, antifungal, antiviral, and antibiofilm activity [19,57]. Exopolysaccharides from plant-derived LAB have been documented to have antimicrobial activity. For instance, Singh et al. [77] isolated *Lactobacillus acidophilus* and *L. bulgaricus* from cabbage and cucumber, and different EPS extracts (80% methanol, 50% methanol, and aqueous) were evaluated for the antibacterial activity (Table 1). Exopolysaccharide derived from *L. acidophilus* showed ZOI with methanol extracts (80% and 50%), and the aqueous extract ranged from 12 to 20.5 mm, from 15 to 21 mm, and from 12 to 18 mm for all tested strains, respectively. MIC values were documented to be 80 μg/mL or higher. Similarly, *L. bulgaricus*-derived EPS displayed ZOI with 80% and 50% methanol, and aqueous extract ranged from 14 to 16 mm, from 9 to 16 mm, and from 10 to 15 mm, respectively, for four tested pathogens (Table 1). MIC values for *L. bulgaricus* were tested to be more than 90 μg/mL [77]. The antibacterial activity of EPS could be attributed to the inhibition of cell wall synthesis, interference with cell membrane integrity, inhibition of protein and nucleic acid synthesis, and inhibition of metabolic pathways [57].

Recently, the promising antiviral application of EPS derived from *Lactiplantibacillus plantarum* has been documented (Table 1). EPS worked against the influenza virus (Alphainfluenza virus Influenza A virus) and feline calicivirus, which is recognized as norovirus. However, the authors have not reported the mode of action of EPS against viruses [78]. It has been described that polysaccharides can suppress viral infections by causing a hindrance to viral absorption in host cells [79]. These studies indicate that EPS not only have well-documented applications in food protection but also act as important antimicrobial agents. More studies are needed to explore EPS from plant-derived LAB as antifungal and anti-biofilm agents as well as against other bacterial and viral pathogens.

Besides antibacterial and antiviral activities, plant-derived LAB also exhibited antifungal activity. The spent media of *Lactiplantibacillus plantarum* KCC-24 isolated from Italian ryegrass unveiled remarkable antifungal activity assessed visually through the pore plate method and using the microdilution method. In the microdilution method, the fermentative metabolites of the tested strain showed a percent (%) inhibition of 59.04 ± 0.74, 56.67 ± 0.72, 40.23 ± 0.43, 52.47 ± 0.68, and 73.43 ± 0.96 for *Penicillium chrysogenum*, *P. roqueforti*, *Botrytis elliptica*, *Fusarium oxysporum*, and *Aspergillus fumigatus*, respectively. The medium without fermentative metabolites was considered as a control. The strongest antifungal activity was reported against *A. fumigatus*. As per the authors, KCC-24 produced a significant amount of organic acids, namely, lactic acid, acetic acid, and succinic acid, which could be the factors behind the remarkable antifungal activity [80]. These studies suggest the antagonistic activity of *Lactobacillus* species against pathogens. Future research should focus on the isolation of potential probiotics from more unconventional sources, their antagonistic effects in vitro and in vivo, the nature of the inhibitory compounds, and in-depth mechanisms of antimicrobial effects.

### 4.2. Leuconostoc

*Leuconostoc* (*Leu*.) species are Gram-positive, non-motile, facultative anaerobes, and catalase-negative LAB. This genus is a member of the Leuconostocaceae family and can be isolated from plants, fermented foods, and dairy sources [81]. The genus has now been designated as GRAS [82]. The common species, *Leuconostoc mesenteroides*, and its postbiotics have been reported to display important antibacterial activity against many foodborne pathogens such as *E. coli*, *Listeria monocytogenes*, *Pseudomonas aeruginosa*, *Salmonella* species, *Staphylococcus aureus*, and *Serratia liquefaciens* [83,84,85,86]. Recent reports have also documented the use of different strains and metabolites of *Leu. mesenteroides* as biocontrol agents against plant pathogens [87,88]. This section describes the antimicrobial activity of different *Leuconostoc* strains derived from plants.

In a study, two different strains (KCC-57 and KCC-58) of *Leu. citreum* isolated from rice plants and their cell-free secondary supernatant (CFS) showed the antibacterial activity against different pathogens, namely *E. coli*, *Enterococcus faecalis*, *Pseudomonas aeruginosa*, and *S. aureus*. The CFS of both strains demonstrated strong ZOI against *E. coli*, trailed by *S. aureus*, *E. faecalis*, and *P. aeruginosa*. Time-killing assay showed that both CFS at 25 mg/mL concentration inhibited all pathogens. Minimum bactericidal activity (MBC) is defined as the minimum concentration of an antibacterial agent needed to kill a particular bacterium. Minimum inhibitory concentration (MIC) of KCC-57 CFS was observed to be 12.5 mg/mL except for *S. aureus*, MIC value was 25 mg/mL, while MBC was reported to be 25 mg/mL against *E. coli* and *P. aeruginosa*. No MBC values were observed for the remaining two pathogens. MIC values for KCC-58 CFS were noted to be 12.5 mg/mL, except for *E. faecalis*, where MIC was observed to be 6.5 mg/mL. Further MBC values were noted to be 25 mg/mL for all pathogens except for *E. faecalis* (12.5 mg/mL). Additionally, a co-culture study revealed that both strains exhibited antagonistic effects via competitive inhibition, suggesting their potential application as biotherapeutic agents in lieu of antibiotics [89]. This study suffers from a limitation that it did not characterize CFS of both strains, as CFS may contain many essential metabolites. Future studies should evaluate the nature and the antagonistic effects of postbiotics from LAB.

Probiotics and various metabolites have been documented to exhibit the anti-listerial activity. As itemized above, the bacteriocin is one of the important antimicrobial agents secreted by probiotic LAB. A study reported the anti-listerial activity of bacteriocins secreted by *Leu. pseudomesenteroides* 607, isolated from the persimmon fruit (Table 1). The researchers purified two antibacterial substances from culture supernatants of strain 607 using chromatographic procedures. One of the peptides, based on its N-terminal amino acid sequences, matches class IIa bacteriocins, noted to be leucocin C based on in silico comparisons. As per the authors, the information on the second peptide (bacteriocin 607A) is still under investigation. ZOI was reported to be 17.5 mm and 14 mm for leucocin C and bacteriocin 607A, respectively [90]. Future research should investigate additional mechanisms by which probiotics and their metabolites exhibit anti-listerial action.

### 4.3. Weissella

Collins et al. [91] first described the genus *Weissella* in 1993 while performing taxonomic studies of a group of *Leuconostoc*-like microorganisms from fermented Greek sausages and proposed reclassifying *Leuconostoc paramesenteroides* and related species into a new genus *Weissella* based on 16S rRNA gene sequence comparisons [91]. To date, 21 *Weissella* species have been documented [92]. *Weissella* isolated from different fermented products have been utilized in the food processing industry for use in food fermentation, EPS production, reduction in chemical constituents, potential antimicrobial activity, and control of pathogens. The presence of *Weissella* species has been documented from many ecological niches such as soil, plants, fruits, and foods [19,93].

The genus has attracted a lot of attention due to its probiotic and biotechnological applications [93]. There have been limited reports on species isolated from plants that exhibit antimicrobial activity. In one study, five *Weissella paramesenteroides* species, FX1, FX2, FX5, FX9, and FX12, isolated from different fruits and their CFS, exhibited antibacterial activity against *E. coli* MTCC1697 and *Staphylococcus aureus* MTCC1144. The growth medium was supplemented with glucose and prebiotics (glucooligosaccharides (GOS) and fructooligosaccharides (FOS)). A higher antibacterial activity against *E. coli* was witnessed after treatment with FOS, followed by glucose and GOS, while for *S. aureus*, higher antibacterial activity was reported for glucose followed by prebiotics (FOS, then GOS). Further, the antimicrobial activity of the extracellular protein concentrate (dialyzed extract with 1:5 dilution) of the selected species FX5 demonstrated 37 ± 2% and 20 ± 1% for *S. aureus* and *E. coli*, respectively. However, the antimicrobial activity of acidic CFS was reported to be higher than that of protein concentrate (dialyzed), implying that the antimicrobial activity was because of the presence of organic acids. This study suggests the prebiotic utilization potential of the selected strains isolated from fruits [94].

### 4.4. Enterococcus

Enterococci are classified as Gram-positive, facultative, anaerobic cocci, which are found in the gastrointestinal tracts of mammals and other animals, water, plants, and soil [95,96]. Despite lacking GRAS approval, certain strains of the genus *Enterococcus* are presently recognized as probiotics and are commercially available [97]. Different *Enterococcus* strains and their metabolites have been used in food preservation and fermentation [98]. *Enterococcus* strains, isolated from plants, are also reported to have antimicrobial activity.

In a study, antimicrobial activity of CFS from *Enterococcus mundtii* ST4V was reported against *E. faecalis*, *Streptococcus* sp., *P. aeruginosa*, *Klebsiella pneumoniae*, *Streptococcus pneumoniae*, and *S. aureus* (Table 1). The enzymatic treatment of the peptide led to a reduction or complete suppression of its antibacterial activity, indicating the presence of a proteinaceous component in CFS. The peptide also exhibited dose-dependent antiviral activity. As per this study, this was the first report pertaining to the broad-spectrum activity of a peptide produced by an *Enterococcus* LAB [99]. Recently, CFS from *Enterococcus mundtii* isolated from parts of a medicinal plant (*Herniaria glabra* L.) showed inhibitory activity against *Bacillus subtilis* and *Klebsiella pneumoniae* and bacteriostatic activity against *Yersinia pseudotuberculosis* (Table 1). The strain also showed potential probiotic properties along with wound healing potential [100]. This study has not evaluated the presence of any active metabolite responsible for the inhibitory action against these pathogens. Previous research reported the role of bacteriocin from *E. mundtii* (isolated from fermented product) as antimicrobial agents [101]. *E. mundtii* from this medicinal plant should be evaluated through genomic and functional characterization for the presence of bacteriocin-producing capacity and potential probiotic traits.

More recently, LAB were screened and isolated from jalapeno peppers (*Capsicum annuum*) that showed antagonistic activity against foodborne pathogens (Table 1). This study assessed the existence of antagonistic compounds in 72 LAB strains and evaluated their heat-resistant capabilities. The antagonistic substance was identified as bacteriocin, as its activity was diminished after treatment with protease. Out of the total, 60 strains retained the selected antibacterial activity. Sixty strains that exhibited sustained activity were further assessed for their heat-resistant capabilities by subjecting them to 121 °C for 15 min. Strain 67 was evaluated for MIC and MBC of bacteriocin-like inhibitory substance against the tested four foodborne pathogens. The MIC (mg/mL) and MBC (mg/mL) values were reported for *E. coli* O157:H7 (250, 400), *L. monocytogenes* (80, 320), *S. aureus* (80, 320), and S. Typhimurium (150, 250) [102]. These findings suggest that future studies should screen more plant-based LAB isolates for the selection of strains with high antagonistic activity against pathogenic and foodborne pathogens. Furthermore, there are concerns regarding *Enterococcus* strains, as some may possess virulence factors [103]. Therefore, further research is needed to assess the occurrence of pathogenic traits [104].

**Table 1:** Antimicrobial activity of LAB strains isolated from different plants and their parts.

Isolation Source	LAB Strains and Products	Main Findings	Ref.
Pulp processing of *Annona muricata* L., *Malpighia glabra* L., *Mangifera indica* L., *Ananas comosus* L., and *Fragaria vesca* L.	*Lactiplantibacillus plantarum* 49, *Levilactobacillus brevis* 59, *Lacticaseibacillus paracasei* 108, *Lactiplantibacillus pentosus* 129, and *Limosilactobacillus fermentum* 111	All strains exhibited antibacterial Activity against *Salmonella* Enteritidis > *Salmonella* Typhimurium > *L. monocytogenes* > *E. coli* > *S. aureus*, based on average ZOI from spot and well-diffusion assays	[75]
Pulp processing of *Annona muricata* L.	*Lactiplantibacillus* *plantarum* 53 and *Lacticaseibacillus* *paracasei* 106	All strains showed antagonistic activity against *Salmonella* Enteritidis, *Salmonella* Typhimurium, *L. monocytogenes*, *E. coli*, and *S. aureus.* Strongest activity was shown by strains 53 and 60	[76]
Pulp processing of *Malpighia glabra* L.	*Limosilactobacillus fermentum* 56, 60		
Pulp processing of *Mangifera indica* L.	*L. fermentum* 139, 141		
Pulp processing of *Ananas comosus* L.	*L. fermentum* 250, 263		
Pulp processing of *Fragaria vesca* L.	*L. fermentum* 296		
Cabbage and cucumber	EPS (80% methanol, 50% methanol, and aqueous extracts) produced by *Lactobacillus acidophilus* and *Lactobacillus bulgaricus*	All extracts exhibit antibacterial activity against *E. coli* (MTCC 1687), *S. aureus* (MTCC 7443), *Salmonella enterica* (MTCC3219), and *Shigella flexeneri* (MTCC 1457) Inhibition by all the extracts *S. aureus* > *S. enterica* > *E. coli* > *S. flexneri*	[77]
Pear	EPS (negatively charged and acidic) produced by *Lactiplantibacillus plantarum* SN35N	EPS suppressed the infectivity of Feline calicivirus (*Vesivirus Feline calicivirus)* and Influenza virus *(Alphainfluenzavirus Influenza A virus)*	[78]
Italian ryegrass (*Lolium multiflorum*) forage	*Lactiplantibacillus* *plantarum* KCC-24	The strain showed antifungal activity against strains of *Aspergillus fumigatus*, *Penicillium chrysogenum*, *P. roqueforti*, *Botrytis elliptica*, and *Fusarium oxysporum* Highest inhibition = *A. fumigatus*	[80]
Rice plants	CFS of *Leu. citreum* KCC-57, KCC-58	KCC-57 (ZOI) *E. coli* = 34.3, *E. faecalis* = 18.5*, P. aeruginosa* = 17.5, and *S. aureus* = 22.0 KCC-58 (ZOI) *E. coli* = 32.6, *E. faecalis* = 22.5, *P. aeruginosa* = 18.5, and *S. aureus* = 27.3	[89]
Persimmon fruit	Bacteriocins (Leucocin C-607 and bacteriocin 607A) of *Leu. pseudomesenteroides*	Bacteriocins inhibited the growth of *Listeria monocytogenes* ATCC 19111	[90]
Orange, sapota, banana, cherry, and plum smashed fruits	CFS and extracellular protein concentrate (FX5) of *Weissella paramesenteroides*	Showed antimicrobial activity against *E. coli* and *S. aureus*	[94]
Soybeans	Peptide from *Enterococcus mundtii* ST4V	Peptide inhibited growth of *E. faecalis*, *Streptococcus spp.*, *P. aeruginosa*, *Klebsiella pneumoniae*, *S. pneumoniae*, and *S. aureus*	[99]
	Peptide concentration (40 and 400 μg/mL)	At 400 μg/mL (% inhibition): HSV-1 (strain F): 99.99% HSV-2 (strain G): 99.98% Measles virus (strain MV/BRAZIL/001/91, an attenuated strain of MV): 95.5% Polio virus (PV3, strain Sabin): 50%	
Leaves, flowers, and roots of *Herniaria glabra* L.	*Enterococcus mundtii*	ZOI (mm, 24, 48, and 72 h) *B. subtilis*: 11–14 mm *Klebsiella pneumoniae*: 9–13 mm *Yersinia pseudotuberculosis:* bacteriostatic activity	[100]
LAB from jalapeno peppers	Different strains of *Enterococcus* *lactis*	Antimicrobial activity of heat-resistant bacteriocin-like component from strain 67 against *L. monocytogenes* ATCC 7644, *S. aureus* ATCC 6538, *E. coli* O157:H7 K3999, and S. Typhimurium	[102]
Assam tea plants (*Camellia sinensis* var. *assamica*)	*B. clausii*, *B. subtilis* ML066-3, *B. licheniformis* ML071-1, ML073-1, ML075-1, ML076-2, and *B. siamensis* ML122-2, ML123-1, ML124-1	Showed activity against *S. aureus* ATCC 25923, MRSA DMST 20625 *B. cereus* TISTR 687, and *E. coli* O157:H7 DMST 12743	[105]
Wild *Bromelia* sp. flowers	*B. subtilis* Fa17.2	Inhibited foodborne pathogens: *S. aureus*, *E. coli*, *Shigella dysenteriae*, and *Kosaconia cowanii*	[106]
Nectar of *Butea monosperma* flower	*Fructobacillus fructosus* MCC 3996	Antagonistic activity against *E. coli* (NCIM 2109), *S. aureus* (NCIM 2079), S. Typhimurium (NCIM 2501), *Proteus vulgaris* (NCIM 2172), *B. pumilus* (NCIM 2327), and *P. aeruginosa* (NCIM 2036)	[107]
Orange juice	*F. tropaeoli* KKP 3032	CFS of KKP 3032 inhibited *E. coli* (KKP 987), *L. monocytogenes* (KKP 1058), *P. aeruginosa* (KKP 994), *S. aureus* (KKP 995), *Salmonella enterica* (KKP 1044), and *B. cereus* (KKP 358)	[108]

Abbreviations: CFS = cell-free supernatant, EPS = exopolysaccharide, MTCC = Microbial Type Culture Collection and Gene Bank, TISTR = Thailand Institute of Scientific and Technological Research, ATCC = American Type Culture Collection, ZOI = zone of inhibition, HSV = herpes simplex viruses, MRSA = methicillin-resistant *S. aureus.*

### 4.5. Bacillus

*Bacillus* strains are Gram-positive, spore-forming, rod-shaped, aerobic or facultative anaerobic bacteria that can be isolated from air, soil, vegetables, fermented foods, animals, and the human gut [109,110]. These probiotic isolates in their spore form are metabolically inactive and can withstand harsh environmental conditions [111]. Many *Bacillus* species have been used as commercial probiotic strains for human use, such as *B. cereus*, *B. polyfermenticus* SCD, *B. clausii*, *B. subtilis*, *B. pumilus*, and mixed type (*B. subtilis* and *B. licheniformis*) [112]. However, special attention should be paid with regard to antimicrobial resistance, toxicogenic potential, and biogenic amine production while investigating their probiotic characteristics [112].

Different *Bacillus* isolates are important pertaining to their health-promoting properties, such as antioxidant, antimicrobial, hepatoprotective, and immunomodulative properties, as well as improvement of the gut environment [113,114,115,116]. Recently, beneficial effects of thermophilic probiotic *Bacillus* isolates have been described owing to the release of various metabolites such as bacteriocins or bacteriocin-like substances, extracellular enzymes, EPS, L-lactic acid, vitamins, amino acids, γ-aminobutyric acid, and other metabolites [117]. Limited literature is available on probiotic *Bacillus* strains isolated from plants and their antimicrobial properties.

Rungsirivanich et al. [105] isolated LAB from Assam tea plants *(Camellia sinensis* var. *assamica)* from six different provinces of Thailand. This study evaluated the antimicrobial activity of culture supernatants of different isolates belonging to the Bacillaceae family. Among all, *B. clausii* ML062-2 was the only isolate inhibiting the growth of *E. coli* O157:H7 with a ZOI value of 7.2 mm. All *B. licheniformis* strains (ML071-1, ML073-1, ML075-1, and ML076-2) and *B. subtilis* ML066-3 displayed antibacterial activity against *B. cereus* TISTR 687 with diameters of inhibitory clear zones ranging from 9.3 mm to 11.3 mm and 7.3 mm, respectively (Table 1). All *B. siamensis* (ML122-2, ML123-1, and ML124-1) strains were evaluated to be positive against methicillin-resistant *Staphylococcus aureus* (MRSA), with ZOI values of 12 mm, 9.3 mm, and 11.3 mm, respectively. The antibacterial activity against *S. aureus* was shown by *B. subtilis* ML066-3 (9.0 mm), *B. licheniformis* ML075-1 (9.0 mm), and *B. siamensis* ML122-2 (8.0 mm). Importantly, among all, the only tested strain that showed growth inhibition of both *S. aureus* and MRSA was *B. siamensis* ML122-2 [105]. This study highlighted the antagonistic activity of some strains against antibiotic-resistant MRSA. This study evaluated the probiotic potential of the promising strains; however, this study has not evaluated the nature of the active compound responsible for the antagonistic activity. Hence, future studies should focus on the compound that led to antagonistic activity and screening of more active and potential LAB strains from different tea variety plants or their parts.

In another study, the crude extract of the potential probiotic strain *B. subtilis* Fa17.2 isolated from a flower showed antagonistic activity against foodborne pathogens, namely *S. aureus*, *E. coli*, *Shigella dysenteriae*, and *Kosaconia cowanii*. This study reported the presence of protein-like an antimicrobial substance in the crude extract. Further analysis confirmed the presence of partially purified bacteriocin-like substances with a molecular weight of 14 kDa [106].

### 4.6. Fructobacillus

Among LAB, a newly discovered and unconventional group is known as fructophilic LAB (FLAB) [118]. These LAB isolates prefer to grow on fructose (unlike conventional counterparts), can be found in fructose-rich niches such as fruits and flowers, and are documented to possess unique biofunctional properties [119]. Until 2008, the genus *Fructobacillus* was thought to be a subgroup of *Leu. fructosum* [118,120]. To date, 11 recognized species of the genus *Fructobacillus* have been reported [121]. Limited literature has been reported on the plant-derived *Fructobacillus* strains and their probiotic and antimicrobial properties. In one study, *Fructobacillus fructosus* was isolated from *Butea monosperma* flower nectar and showed antagonistic activity against *E. coli*, *Bacillus pumilus*, S. Typhimurium, *S. aureus*, *Proteus vulgaris*, and *P. aeruginosa.* As per authors, the antagonistic activity could be due to components such as organic acids or H_2_O_2_, etc., as the selected strain lacks the ability to produce bacteriocin [107]. More recently, another species, *F. tropaeoli* (KKP 3032), isolated from orange juice, was assessed for its probiotic properties, including antagonistic activity against foodborne pathogens. The highest (26.3 mm) and lowest (15.7 mm) inhibition zones by KKP 3032 CFS were observed against *L. monocytogenes* (KKP 1068) and *Bacillus cereus* (KKP 358), respectively. However, this study has not evaluated the nature of the antibacterial compound present in CFS [108].

### 4.7. Multi-Strain LAB Isolations

Different studies have reported the isolation of two or more LAB species from different parts of plants, fresh vegetables, and fruits and evaluated them for their antagonistic activities against pathogens, as presented in Table 2.

Samedi et al. [122] screened five different LAB strains from the leaf surface of cassava, papaya, sugarcane, taro, and yam and demonstrated antagonistic activity against Gram-positive and Gram-negative foodborne bacteria (Table 2). *Weissella* (*W.*) *paramesenteroides* C04, *E. faecalis* S02, *L. paraplantarum* P01, *L. plantarum* T03, and *W. paramesenteroides* Y05 exhibited different levels of the antibacterial activity. In particular, isolate P01 with ZOI more than 9 mm displayed greater and stronger activity against *E. coli* and *S. aureus* compared to other strains. Strains C04 and Y05 did not exhibit activity against *B. cereus*. Overall, ZOI from different strains ranged from > 3 mm to > 9 mm [122]. This study indicates that LAB isolate type and isolation source might have impacted the functional properties of different LAB strains. 

As stated above, H_2_O_2_ production has been observed to be responsible for the antimicrobial activity of LAB isolates. On this line, dos Santos et al. [123] reported the production of H_2_O_2_ and ZOI (halo, mm) against seven pathogenic and spoilage microorganisms (Table 1). A total of thirteen strains belonging to the genus *Lactobacillus* and *Pediococcus* showed antimicrobial activity. Zones of inhibition ranged from 13 to 28 mm, from 11 to 24.67 mm, from 15.33 to 32.00 mm, from 10.33 to 20.00 mm, from 15.00 to 32.00 mm, from 1.00 to 20.00, and from 6.33 to 29.67 against *Staphylococcus* sp., *Proteus mirabilis, Salmonella* sp., *E. coli, Pseudomonas* sp., *Shigella* sp., and *Klebsiella* sp., respectively. Among all LAB, two *plantarum* strains (SBR64.2 and SBR64.12) isolated from grass silage exhibited the highest antimicrobial activity (against *S. aureus*, *Klebsiella* sp., and *Pseudomonas* sp., and against *Salmonella* sp., *S. aureus*, and *Pseudomonas* sp., respectively) (Table 2). As per this study, the production of H_2_O_2_ was not the sole criterion for antimicrobial action, as some strains did not produce it and still demonstrated the antimicrobial activity. Thus, the antimicrobial activity of non-H_2_O_2_ LAB strains could be due to the presence of other compounds such as organic acids or bacteriocins [123].

Over the past two decades, opportunistic fungal species, particularly *Aspergillus* spp. and *Candida* spp., have emerged as established pathogens. Compared to the chemical-based drugs, probiotics (*Lactobacillus*, *Leuconostoc*, and *Saccharomyces* spp.) and their metabolites have emerged as natural and environmentally safe antifungal alternatives. Mechanistically, they work based on competitive exclusions and immunomodulation. The infections by *Aspergillus* species can result in significant morbidity and mortality [124]. Limited information is available on the antifungal activity of LAB isolates derived from plants. In one study, LAB strains *Lactiplantibacillus plantarum* (RG7B) and *Pediococcus pentosaceus* (RG7B and C11C) from two grape varieties showed antifungal activity against ochratoxin A-producing *Aspergillus niger* aggregates and *Aspergillus carbonarius* strains [125]. The ochratoxin family has more than 20 subtypes and is a noted human carcinogen that has hepatotoxic, nephrotoxic, mutagenic, teratogenic, and immunosuppressive effects [126,127].

Junnarkar et al. [128] evaluated the antibacterial activity of CFS against potential human pathogens from 25 LAB strains (*Lactobacillus* sp., *Enterococcus* sp., and *Weissella* sp.) isolated from fresh vegetables (Table 2). The ZOI against six human pathogens ranged from 10 to > 20 mm. Maximum inhibition of *Citrobacter freundii* was noted from *Enterococcus* strains (ID8V and ID11V) isolated from fenugreek. *Citrobacter freundii* is reported to be an opportunistic pathogen and an agent of nosocomial infections [129]. Isolates from tomatoes, both *Lactobacillus* sp. (J129V, J131V), were reported to be most potent against tested pathogens. A few *Lactobacillus* and *Enterococcus* isolates did not show activity against selected pathogens. It could be due to the fact that functional properties of LAB are strain-specific features and are affected by genetic variations, isolation sources, time of isolation, and the geographical location. This study suggested the role of bacteriocin and bacteriocin-like substances as potential antimicrobial substances [128].

**Table 2:** Antimicrobial activity of multi-lactic acid bacteria (LAB) strains isolated from different plants and their parts.

Isolation Source	LAB Strains	Main Findings	Ref.
Fresh papaya leaves	*Lactiplantibacillus paraplantarum* P01	All strains showed antagonistic activity against *E. coli*, *B. cereus*, *L. monocytogenes*, and *S. aureus*	[122]
Yam	*Weissella (W.) paramesenteroides* Y05		
Taro	*Lactiplantibacillus plantarum* T03		
Sugarcane	*E. faecalis* S02		
Cassava	*W. paramesenteroides* C04		
Grass silage	*Lactiplantibacillus plantarum* SBR64.1, SBR64.2, SBR64.5, SBR64.7, SBR64.12	Antagonistic activity against pathogenic and spoilage microorganisms: *E. coli, Klebsiella* sp., *Pseudomonas* sp., *Staphylococcu*s sp., *Shigella* sp., *Salmonella* sp., and *Proteus* *mirabilis*	[123]
Alfalfa silage	*Lactiplantibacillus pentosus* SA64.2 *P. acidilactici* SA64.6		
Elephant grass silage	*Lacticaseibacillus paracasei* SCE50.5		
Peanut silage	*Lacticaseibacillus zeae* SAM50.5		
Sorghum silage	*Lentilactobacillus buchneri* SS50.1, SS50.4 *Limosilactobacillus fermentum* SS50.9, SS50.10		
Two grape varieties (cardinal and red globe)	*P. pentosaceus* RG7B, C11C *Lactiplantibacillus plantarum* RG8A	RG7B, C11C, and RG8A showed antifungal activities against *Aspergillus niger* and *A. carbonarius*	[125]
Fresh vegetables (French beans, cauliflower, gherkins, fenugreek, bitter gourd, cluster beans, tomato, ridged gourd, and bottle gourd)	Twenty-five LAB isolates: *Lactobacillus* sp. LAB8V, LAB6V, J122V, J129V, J131V, ID12V, ID7V, ID13V and others *Enterococcus* sp. ID8V, ID11V, ID18V, ID19V, and AGIV *Weissella* sp. ID10V	Cell-free supernatant (CFS) produced bacteriocin- and bacteriocin-like substances. Extracts showed activity against human pathogens: *E. coli*, *K. pneumoniae, Staphylococcus epidermidis*, *B. cereus*, *Citrobacter freundii*, and *Enterobacter cloaceae*	[128]
Fresh fruits (banana, Chinese peach, and kiwi fruit) and flowers (narcissus, pink rose, yellow rose, and sunflower)	*Fructobacillus pseudoficulneus* JNGBKS, JNGBKS3, *F. fructosus* JNGBKS2, JNGBKS4, *F. durionis* JNGBKS5, and *Lactobacillus kunkeei* JNGBKS6, JNGBKS7, JNGBKS8	The fructophilic LAB strains inhibited *E. coli*, *S.* Typhimurium, and *S. aureus* pathogens	[130]
Corn stover silage	*Lactiplantibacillus plantarum* subsp. *plantarum* ZZU 204, 273, 274, 278, 203 283, and 299, *P. pentosaceus* ZZU 64, 223, *E. mundtii* ZZU 205, *W. cibaria* ZZU 50, and *Leu. pseudomesenteroides* ZZU 170	Isolated LAB species inhibited *Salmonella enterica*, *Micrococcus luteus*, and *E. coli*	[131]
Cilantro and cantaloupe melons	*P. pentosaceus* CM175 and *Latilactobacillus graminis* C15	CFS displayed antagonistic activity against S. Typhimurium, *Salmonella* Saintpaul, *S. aureus*, *L. monocytogenes*, and *E. coli* O157:H7	[132]
*Donax canniformis*, *Dysoxylum parasiticum*, *Tabernaemontana aurantiaca*, *Ficus arfakensis*, *Galearia celebica*, *Pinanga* sp., *Lasianthus* sp., *Dracaena angustifolia*, and *Myristica subalulata*	*Lactococcus lactis* HM1.1 HM1.2, H12.1, H12.2, HM7, H10.1, H10.2, H3.1, and H3.2	All isolated strains displayed antagonistic activity against *E. coli* InaCC B5, *Mycobacterium smegmatis* NBRC 3082, and *S. aureus* InaCC B4	[133]
*Capparis* sp.	*W. confusa* H14.2		
*Syzygium* sp.	*Lactococcus garvieae* H9.1		
*Tetrastigma papillosum*	*Enterococcus faecalis* H4.1		
*Cordyline* sp. and *Helicia moluccana*	*W. oryzae* H13.2, H11.2		
Bell pepper	*Leu. mesenteroides* PIM5	CFS displayed antimicrobial activity against Gram-positive bacteria (*B. cereus*, *L. monocytogenes*), LAB (*L. lactis*, *L. casei*), molds (*A. niger*, *F. oxysporum*, and *P. expansum*), and yeasts (*Candida albicans*, *C. tropicalis,* and *S. cerevisiae*). No activity against Gram-negative bacteria	[134]
Zucchini	*Leu. mesenteroides* CAL14		
Tangerine	*Leu. mesenteroides* MAD3		
Guava	*Leu. mesenteroides* GUA13		
Cucumber	*Leu. mesenteroides* PEP12		
Cucumber	*Enterococcus faecium* PEP11		
Bell pepper	*E. faecium* PIM4		
Corn	*Enterococcus mundtii* ELO8		
G. tomato	*E. mundtii* TOV9		
Orange	*E. mundtii* NAR1		
Red apple	*E. mundtii* MR15		
Jalapeño	*E. mundtii* JAV15		
Açai fruits	*Lactiplantibacillus plantarum* B144, B143, B142, B141, B140, B135, B150, Z183, and Z170 and *P. pentosaceus* C21, B134, B125, C52, B139, B137, B109, B113, and B138	Twenty-seven strains showed antagonistic activity against *E. coli*, S. Typhimurium, *E. faecalis*, and *S. aureus*. Strain C52 had no activity against *E. coli*	[135]
Bacupari-do-cerrado, gabiroba (M1, M2), guapeva, pequi peel (M1, M2, M3), pequi mesocarp (M1, M2, M3), mangaba, and puç’a (M1, M2, M3)	*Lactiplantibacillus plantarum*, *L. pentosus*, *Lacticaseibacillus casei*, *Lacticaseibacillus paracasei*, *P. acidilactici*, *W. cibaria*, and *W. confusa*	Eleven isolates showed activity against *E. coli*, *S. aureus*, and *Salmonella* sp.	[136]
Tomato, peach, cucumber, strawberry, cabbage, lettuce, and parsley	*Enterococcus faecium* F1, F13, F15, F18, F25, F31, and F37, *E. durans* F23, F26, F40, F41, and F43, *E. faecalis* F46, *E. lactis* F8, *P. acidilactici* F21, F28, Both heat-killed and probiotic LAB	Both LAB and heat-killed bacteria inhibited growth of *E. coli*, *S. aureus*, *S.* Typhi, and *L. monocytogenes*	[137]

With regard to the genus *Fructobacillus*, Sakandar et al. [130] screened FLAB from Chinese flowers and fruits for probiotic and antibacterial potential and selected eight strains belonging to *Fructobacillus fructosus*, *Lactobacillus kunkeei* (now known as *Apilactobacillus kunkeei*), *F. pseudoficulneus*, and *F. durionis.* The antagonistic activity exhibited ZOI in the range of 6.5 to 9.5 mm, 6.0 to 8.5 mm, and 4.0 to 7.5 mm against *E. coli*, *S. aureus*, and S. Typhimurium, respectively (Table 2). Among all, *L. kunkeei* showed maximum inhibition of all pathogens, while *F. durionis* showed the lowest inhibition. This study suggested the potential involvement of bacteriocin metabolites as anti-pathogenic agents [130]. More studies are needed, especially on probiogenomics in the near future, to ascertain reasons for differences in probiotics and functional properties between FLAB and conventional LAB.

Lactic acid bacteria isolated from corn stover silage displayed antagonistic activity against *Salmonella enterica*, *Micrococcus luteus*, and *E. coli*, with more than 8 mm ZOI. Moreover, among tested strains, two *Lactobacillus* strains (ZZU 203, ZZU 204) demonstrated strong antibacterial activity even after neutralizing the effects of organic acids and hydrogen peroxide from CFS. However, the antibacterial activity vanished fully following the treatment of CFS with proteinase K. These findings indicated the proteinaceous nature of the antimicrobial compound. Further, after trypsin treatment, the antibacterial activity of the strain ZZU 203 vanished, while ZZU 204 still exhibited activity against *E. coli* and *M. luteus*. As per the authors, the difference in the antibacterial activity could be due to the different mode of action of proteinase K and trypsin [131]. From another study, *P. pentosaceus* (CM175) and *Latilactobacillus graminis* (C15) isolated from cantaloupe melons and cilantro exhibited antagonistic activity against foodborne microorganisms (Table 2). Except for *E. coli*, CFS from both strains (CM175, C15) showed antibacterial activity with ZOI ranging from 6 to 17 mm and from 4.33 to 5 mm, respectively. CM175 displayed the higher growth inhibition potential, while C15 CFS displayed a bacteriostatic effect. The presence of organic acids and bacteriocin-like components was noted to be responsible for the antagonistic activity of LAB strains.

Dinoto et al. [133] isolated LAB from Indonesian plants belonging to 14 different families and screened them for their antibacterial activity against *Mycobacterium smegmatis*, *S. aureus*, and *E. coli* (Table 2). *Mycobacterium smegmatis* is a biofilm-synthesizing bacterium that is not pathogenic to mammals and serves as an alternate model organism in studies on *Mycobacterium tuberculosis* [138]. Among all, HM.1.1 (from *Donax canniformis*) and HM 14.2 (from *Capparis* sp.) strains strongly inhibited *M. smegmatis* with 4.5 mm and 2.0 mm and *S. aureus* with 2.0 mm and 1.5 mm inhibition zones, respectively. Further analyses showed that the MIC values of culture supernatants were lower than those of commercial probiotic strains [133].

Similarly, from another study, LAB isolated from fresh vegetables and fruits showed the potential for antimicrobial activity. LAB isolates with the best activities were molecularly identified as *Leu. mesenteroides*, *Enterococcus mundtii*, and *Enterococcus faecium* [134] (Table 2). Abe Sato et al. [135] reported the antagonistic activity of 28 LAB strains isolated from acai fruits against *E. coli*, S. Typhimurium, *E. faecalis*, and *S. aureus*. Except for one strain, all strains showed the antagonistic activity (Table 2). Among all, the B135, B134, and B125 strains showed the highest antibacterial performance. Against *E. coli*, two strains (Z183 and C21) and against *E. faecalis*, strain B141 showed the highest inhibitory activity. With respect to *S. aureus*, eight strains (B135, Z188, B125, A74, B134, B142, B143, and Z190) exhibited superior antibacterial activity. Compared to the reference strain, nine strains (B109, C39, A71, B113, B134, B140, B135, C37, and B125) exhibited a higher inhibitory capacity against S. Typhimurium. This study also reported the application of the antibacterial activity of açai-derived LAB in açai juice contaminated with two of the four test pathogens, *E. coli* and S. Typhimurium. In this study, the authors discussed the potential roles of bacteriocin and organic acids as potential antimicrobial compounds, but no analysis was performed to identify the nature of the antibacterial compound. Future studies should compare açai fruit-derived LAB isolates from different geographical locations for their probiotic and antimicrobial activities [135].

A separate study isolated LAB from leftovers (peels and seeds) of different fruits grown in ecoregions of tropical savanna (Brazilian Cerrado). Out of 14, 11 LAB strains showed antibacterial activity against the three tested pathogens (Table 2). As per this study, the antagonistic effects were not due to the competitive exclusion as the LAB strains were initially subjected to a short-term treatment with chloroform and UV irradiation. The authors suggested that the plausible nature of the compounds could be bacteriocin, organic acids, or phenolic compounds [136]. These compiled studies suggest that parts of plants serve as an excellent source for potential probiotic strain isolation bestowed with desirable functional properties. Nonetheless, these characteristics are peculiar to particular strains; hence, further investigation is necessary to identify the probiotic strain with beneficial health benefits. Despite the myriad advantages, concerns have been raised regarding the negative effects of probiotics, especially in those with an impaired gastrointestinal mucosal barrier, immunocompromised people, patients recovering from surgery, and premature neonates [139]. Hence, researchers are evaluating postbiotics and paraprobiotics for their health-promoting properties. For instance, Alameri et al. [137] compared the antimicrobial activity of LAB and their heat-killed forms against *E. coli*, *S. aureus*, *Salmonella* Typhi, and *L. monocytogenes*. Both probiotics and heat-killed bacteria inhibited the selected pathogens at different levels. The authors isolated LAB from fruit and vegetable products (Table 2). This suggests that both probiotics and postbiotics should be assessed for their antimicrobial properties against various human and foodborne pathogens. Aside from that, more research is needed to examine the synergistic antibacterial activity of various probiotic LAB against other microorganisms, including pathogens.

## 5. Challenges and Regulatory Considerations

Plant-derived LAB and derived by-products can be utilized in diverse food applications, probiotic formulations, or medical purposes. The antimicrobial efficacy of probiotic LAB is a well-known yet underexplored research domain, laden with substantial challenges.

Most of the studies focus on the probiotic potential of plant-derived isolates and their antibacterial properties in vitro. The genetic composition and physiology of the examined strain are critical factors influencing the antibacterial action. Furthermore, the absence of standardization and inconsistent experimental circumstances may result in conflicting results.

Information pertaining to in vivo uses is scarce. Consequently, subsequent research should examine the antimicrobial efficacy utilizing animal models. Regrettably, certain bacterial and viral pathogens lack specificity for animal models, as they are primarily tailored to human tissues. This is because of the structural and functional variations of the intestinal epithelial tissues [140].

The efficacy of probiotics depends upon the exact bacterial strain or mixture of strains employed for the prevention and treatment of a particular ailment. The application of probiotic organism-based microbial preparations in medicine is impeded by challenges in standardization and the establishment of suitable protocols to maintain the beneficial qualities of microorganisms during the production process [141].

Research on probiotic bacteria must be meticulously directed throughout the entire process until it reaches the clinical phase. As for now, there is no consensus or standardization on the clinical application of probiotics as an antibacterial medicine. Further, the doses of the producer strains, their complex mode of action, commercial viability, and clinical efficacy have yet to be established [20]. The efficacy and safety of medications derived from probiotic strains, together with their advertising costs, are heavily influenced by the level of statutory and technical regulation in the market [141].

Further concern is the acquisition of drug resistance by certain probiotics via various techniques coupled with limited knowledge of transferable resistance among probiotic LAB strains, highlighting a serious safety issue. A recent analysis based on 1901–2022 data pertaining to antimicrobial resistance genes in 12 commonly employed probiotics (579 isolates) revealed the presence of mobile resistance genes in eight bacterial species [142]. Consequently, genome-level screening of mobile drug resistance genes, in conjunction with other virulence factors in novel strains, should be essential [143]. Furthermore, important guidelines must be established to monitor these mobile elements [142].

## 6. Conclusions

The review paper presents comprehensive information on the probiotic LAB strains (*Lactobacillus*, *Bacillus*, *Fructobacillus*, *Pediococcus*, *Leuconostoc*, *Enterococcus*, and *Weissella*) isolated from unconventional sources, including plants, fresh fruits, fruit juices, fresh vegetables, roots, and flowers, along with their functional antimicrobial potential against human pathogens and foodborne microorganisms. The antimicrobial efficacy against pathogens is regarded as a crucial factor in the selection of a potential probiotic strain. The crude extract, postbiotic components, and paraprobiotic of probiotic bacterial strains exhibited potential antibacterial, antifungal, and antiviral properties. The compiled studies showed that bacteriocin and bacteriocin-like substances remarkably contribute to the antimicrobial activity of the presented probiotic strains. Nonetheless, other studies have documented the involvement of several non-bacteriocin components, including peptides, organic acids, EPS, H_2_O_2_, and reuterin, as antimicrobial agents.

Probiotics could inhibit pathogens through competitive exclusion, secretion of metabolites, and interference with the biofilm formation. Still, additional research should be aimed at identifying new molecules and the molecular mechanism of pathogen inhibition comprehensively.

With limited data, the review also highlighted the importance of postbiotics and paraprobiotics as effective antimicrobial strategies. The survival of free probiotic cells in commercial products is a major challenge. Postbiotics, as non-living molecules, have advantages over probiotics; hence, the establishment of standardized protocols exhibiting antibacterial activity is an attractive option [144]. Another solution to the low survival rate of free probiotic cells is to provide living cells with a physical barrier to withstand harsh environmental conditions via microencapsulation. Nonetheless, the production of sturdy capsules that can endure prolonged periods of continuous use without degradation of probiotic cellular activity or capsule properties continues to pose challenges for the industrial application of encapsulated cells. Thus, future studies could use nanoencapsulation technologies for probiotic free cells and other components for protection against environmental factors. However, the risks of nanomaterial use for humans need to be studied and explored extensively [145].

Moreover, there is a continuous search for a novel probiotic strain exhibiting enhanced health-promoting capabilities, and the microbiome linked to plant-derived microenvironments offers an innovative and sustainable resource [146,147]. Most of the presented studies assess the antimicrobial activity of CFS in vitro that can be affected by the composition of the culture media, incubation time and temperature, pH, and the interaction of the tested LAB with other microorganisms present in the same ecosystem. The implementation of in silico tools such as heat maps, principal components, and network analyses for the selection of potential probiotic strains has captured the attention of the researchers [148]. These tools should be utilized frequently for the identification of potential probiotic LAB isolates.

In summary, plant-derived LAB strains exhibit antibacterial, antifungal, and antiviral activities. Nonetheless, the consensus regarding the application of probiotic LAB in vivo and clinically, including their dosages, detailed mechanisms of action, and clinical safety, remains to be determined.

## Data Availability

No new data were created or analyzed in this study.
